# Shedding Light on the Shadows: Hidden Diversity in the Italian Embioptera

**DOI:** 10.3390/insects15110868

**Published:** 2024-11-05

**Authors:** Fabio Cianferoni, Filippo Ceccolini

**Affiliations:** 1 Research Institute on Terrestrial Ecosystems (IRET), National Research Council of Italy (CNR), Via Madonna del Piano 10, I-50019 Sesto Fiorentino (Florence), Italy; 2 Zoology, “La Specola”, Natural History Museum, University of Florence, Via Romana 17, I-50125 Florence, Italy; 3 Via Europa 16/A, I-52016 Rassina (Arezzo), Italy; ceccolinif@virgilio.it

**Keywords:** Calabria, *Embia*, *Haploembia*, new species, Tuscany, Ventotene, webspinners

## Abstract

In order to better understand the differences at the species level in webspinners (Embioptera), the authors applied a different approach rather than the usual slide mounting, which has been traditionally used in the study of these insects. The dissection of the male terminalia, besides avoiding the limits of definitive preparations, which prevent a complete comparison of the type material, allowed a complete understanding and comparison of these three-dimensional structures. This new method, which does not necessarily exclude the previous approach, allowed the authors to identify and describe three new species in Italy: from Tuscany (*Embia specolensis* sp. nov.), Ventotene Island (*Embia pandateriensis* sp. nov.), and Calabria (*Embia brutia* sp. nov.). Moreover, this charts a new path in the study of these interesting insects, which have so far been largely neglected. In addition to several new records resulting from the revision of some collections, this contribution provides an updated checklist and a provisional key for the Italian Embioptera.

## Simple Summary

A new integrative approach to the study of Embioptera is adopted and discussed. It is essentially based on the dissection and three-dimensional observation of pieces of the male terminalia from different angles. This method allows a comprehensive comparison of specimens and evaluates their differences and similarities without the two-dimensional limits (and definitive preparation) of slide mounting. Three new species from Italy are described and several new records are also given. An updated checklist and a provisional dichotomous key for the Italian Embioptera are provided.

## 1. Introduction

Webspinners (Embioptera) constitute a small order of gregarious insects mainly widespread in tropical areas, including less than 400 known species [1]. They live in tunnels built by themselves in lucifugous and hygrophilous habitats, spinning silk from unicellular glands occurring in their enlarged foreleg basitarsi. They often exhibit subsocial behavior. The female and its offspring live together in a network of interconnected silk tunnels [2,3,4]. They are phytophagous, even if, in some species, the female eats the male during mating [5,6].

They are classified into the Polyneoptera, a sister group of the Phasmatodea, according to the recent phylogenies provided by Miller et al. [3] and Wipfler et al. [7].

The real number of living species is certainly underestimated since the Embioptera are among the most neglected groups of insects [8,9]. In Europe and the Mediterranean basin, these insects were very poorly studied. The main contributions were those by Stefani [10,11,12]—who also published many articles about the biology of some species, mainly *Haploembia solieri* Rambur, 1842 e.g., [13,14,15,16,17]—and Ross [18,19] before the group was “rediscovered” in some recent papers by the Italian entomologist Paolo Fontana and colleagues.

In Italy, nine species are currently recorded [20,21,22]. After the five species included in the checklist by Failla et al. [23], in recent years, four more were added by Fontana [21,24,25] and Fontana and Forbicioni [22]. The remark below concerns a further species—*Haploembia tarsalis* (Ross, 1940).

In Italy, the most represented genus is *Embia* Latreille, 1829. The first species recorded in this country was *E. ramburi* Rimsky-Korsakow, 1905—a type locality in France according to Rimsky-Korsakow [26]—which Silvestri [27] cited as collected around Rome in unspecified years before 1948. Currently, this species is reported in different localities in Italy, including Sardinia and Sicily [20]. The immature specimens collected in central Sardinia mentioned by Krausse [28] in the description of *E. kraussi* Krausse, 1911 were subsequently identified by Krausse himself [29] as specimens of *E. ramburi* (synonymizing *E. kraussi* with *E. ramburi*).

Two new species from Sardinia were described by Stefani [10]: *E. tyrrhenica* Stefani, 1953 and *E. nuragica* Stefani, 1953. The latter is considered an endemism of Sardinia and Sicily, whilst *E. tyrrhenica* is reported in some regions of Continental and Peninsular Italy, in addition to Sardinia and Sicily [20].

Silvestri [27] also reported another species in Italy. He identified as *E. savignyi* Westwood, 1837 some specimens collected by himself and other entomologists in Rome and its surroundings, but it is considered a misidentification and the specimens were subsequently attributed by Stefani [10] to *E. tyrrhenica* Stefani, 1953; *E. savignyi* is not considered part of the Italian fauna anymore [20,23].

Finally, in the last two decades Fontana [24,25] described two other species, *E. girolamii* Fontana, 2001—from the Tyrrhenian coast of Tuscany and *E. cynthiae* Fontana, 2002—from Sardinia, and very recently *E. minapalumboi* Fontana, 2024 from Sicily [21] and *E. ilvana* Fontana and Forbicioni, 2024 from Elba [22].

## 2. Materials and Methods

The material examined is ordered as follows: administrative divisions (from largest to smallest: region, province, municipality; approximately ordered from north to south and from west to east), locality, elevation (meters above sea level), georeferencing (geographic coordinates in decimal degrees; datum: WGS84), date, collector(s), number of specimens, sex and wing morph, repository, verbatim transcription of the original label (in quotation marks; new lines are separated by a vertical bar and the different labels are separated by a double vertical bar). Inferred data are enclosed in square brackets. The uncertainty of the georeferenced sites (in meters) is indicated according to the point-radius method [30].

Images of the habitus and terminalia of the specimens were taken with a Leica M205 C stereomicroscope (Leica Microsystems Inc., Deerfield, IL, USA) and dedicated software (Leica LAS v. 4.3) for Z-stacking at the Natural History Museum of the University of Florence. Some of the specimens were examined with a Nikon Eclipse Ci-L biological microscope and Nikon CFI Plan Achromat 4×, 10×, and 20× objectives (Nikon Instruments Inc., Melville, NY, USA). Post-processing of the figures was performed with Adobe Photoshop CS3 Extended (v. 10.0). Measurements were taken with a micrometer eyepiece and ImageJ (v. 1.53).

The body length is very variable in mounted specimens, especially according to how stretched the abdominal segments are (but there may also be significant differences in specimens preserved in ethanol). For the body length (measured from the apex of the mandibles to the apex of the terminalia, i.e., LC_2_), the precision is given to one decimal place. Two decimal places are given for the cranium (measured from the apex of the mandibles to the posterior angles), terminalia and wing measurements.

The term medium-sized [18] is used for all our species that have a body length ranging between ca. 7.5 and 13 mm and are comparable in size to the Mediterranean specimens treated by the same author [18].

The terminology and abbreviations used are according to Ross [18] (p. 9) = ninth abdominal tergite; 10 L = left hemitergite of the tenth segment; 10 LP = process of 10 L; 10 R = right hemitergite of the tenth segment, H = hypandrium, or ninth abdominal sternite; HP = process of H; LC_1_ = basal segment of the left cercus; LC_2_ = apical segment of the left cercus; LPPT = left paraproct; LPPT P = process of the left paraproct; MF = median flap of 10 R; EP = epiproct.

Collection acronyms:

CEM = Collection Enrico Migliaccio; preserved in MZUF.

CFCe = Collection Filippo Ceccolini, Rassina (Arezzo), Italy.

CFCi = Collection Fabio Cianferoni, Florence, Italy.

CFG = Collection Francesca Graziani, Florence, Italy.

CNRF = Invertebrate collection of the National Research Council of Italy (CNR), Research Institute on Terrestrial Ecosystems (IRET), Florence; currently hosted in MZUF.

MZUF = Natural History Museum of the University of Florence (general entomology collection), Florence, Italy.

Other abbreviations used in the text: apt. = apterous; brach. = brachypterous; macr. = macropterous; un. = uncertainty.

### 2.1. Species Concept

The species concept used in this paper follows the criteria traditionally used in the study of Embioptera. According to Ross [18], the most important characteristic in the identification of webspinners’ species is the morphology of the terminalia and, in particular, the process of the left hemitergite of the tenth segment (10 LP), the process of the left paraproct (LPPT P), and the basal segment of the left cercus (LC_1_). These structures are involved in mating; therefore, they are presumed to be species-specific, and they can be used quite confidently for the recognition of differences between species that show a very similar habitus. In the literature, many species were also described based on a single male specimen, e.g., see [18,21,22,24,25], due to the difficulty of finding many specimens.

### 2.2. A New Integrative Approach Used in the Study of Specimens

The male terminalia were dissected under a stereomicroscope. We decided not to clear the pieces in a KOH solution and mount them on slides (see, e.g., [9]) since we wanted to be able to examine the three-dimensional pieces from different points of view under a stereomicroscope and not just in two-dimensional vision. The various pieces were glued (with water-soluble glue) onto a card with the specimen and then examined and photographed.

## 3. Results

### 3.1. Taxonomic Treatment

**Family Embiidae Burmeister, 1836**
**Genus *Embia* Latreille, 1829**

**Diagnosis.** Adult and immature specimens with one ventral papilla on the hind basitarsus. Adult males apterous or winged. Basal segment of the left cercus (LC_1_) with a simple inner lobe; echinulate on the inner surface.

**Figure 1:**
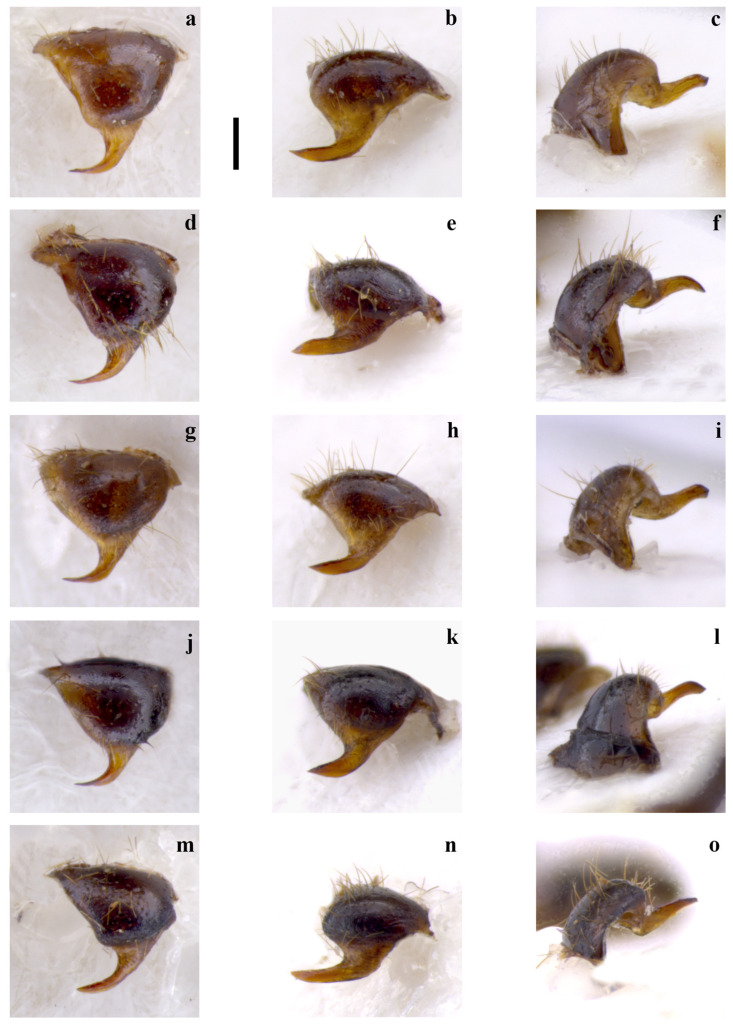
Left hemitergite of the tenth segment (10 L) in the dorsal (**a**,**d**,**g**,**j**,**m**), process of the 10 L (10 LP) from below (**b**,**e**,**h**,**k**,**n**) and from the inner side (**c**,**f**,**i**,**l**,**o**) of *Embia tyrrhenica* Stefani, 1953 (**a**–**l**) and *E. panda-teriensis* sp. nov. (**m**–**o**). Scale bar = 0.2 mm. Photos by Fabio Cianferoni.

***Embia tyrrhenica*** **Stefani, 1953**
  (Figures 1a–l, 3a–h, 4a–d, 5a–d, 7a and 9s,t)

*Embia tyrrhenica* Stefani, 1953. Ross [18] (p. 296); Fontana [25] (p. 42, *partim*); [31] (p. 540)
  *Embia savignyi* Westwood, 1837. Silvestri [27] (p. 13)—suspected misidentification.

**Diagnosis.** Medium-sized dark *Embia*, with males (usually macropterous) characterized by 10 LP abruptly curved before the apex and folded inward, LPPT P claw-like, and LC_1_ with pronounced curvature, starting abruptly near the half of it.

**Material examined.** Tuscany: Florence Metropolitan City, Sesto Fiorentino Municipality, National Research Council of Italy, Florence headquarters, [43.818972° N 11.200792° E; uncertainty = 30 m], [39 m a.s.l.], under a bat colony (*Pipistrellus kuhlii*), V.2023, L. Ancillotto leg., 1 ♂ macr., CNRF; *idem*, 1 ♂ macr., CFCi, *verbatim labels:* “Sesto Fiorentino, sede CNR-IRET, sotto colonia *Pipistrellus kuhlii*, maggio 2023, Leonardo Ancillotto”; Florence Metropolitan City, Florence Municipality, Florence, Via del Poggio Imperiale, 43.757909° N 11.243155° E (uncertainty = 3 m), 66 m a.s.l., vagrant on a sandstone sidewalk, 12.VI.2024, F. Cianferoni leg., 1 ♂ macr., CFCi, *verbatim labels:* “Toscana, Firenze, Via del Poggio Imperiale, 43.757909° N 11.243155° E (incertezza = 3 m; WGS84), 66 m s.l.m., vagante su marciapiede in arenaria, 12.VI.2024, F. Cianferoni leg.”. Lazio: Rome Capital Metropolitan City, Monterotondo Municipality, Monterotondo, in the city, [42.05468° N 12.62036° E; uncertainty = 1500 m], [70–150 m a.s.l.], 3.VI.1989, E. Migliaccio leg., 1 ♂ macr. (Figure 7a), CEM, *verbatim labels:* “Lazio (RM) | Monterotondo in | città 3.VI.1989 | leg. E. Migliaccio || ex collezione | Migliaccio | n° Mag. 3117”; Rome Capital Metropolitan City, Rome Municipality, Tenuta dell’Insugherata [Insugherata estate, today in part a natural reserve], [41.95953° N 12.42607° E; uncertainty = 2200 m], [50–135 m a.s.l.], 29.VI.1942, A. Cotta leg., 1 ♂ macr., CEM, *verbatim labels:* “Roma | Tenuta Insugherata | 29.VI.42 | A. Cotta || ex collezione | Migliaccio | n° Mag. 3117”. Frosinone Province, Filettino Municipality, Filettino, [41.89062° N 13.32439° E; uncertainty = 900 m], [915–1095 m a.s.l.], 28.VI.1981, E. Migliaccio leg., 1 ♂ macr., CEM, *verbatim labels:* “Lazio | Filettino | 28.VI.81 | leg. Migliaccio || ex collezione | Migliaccio | n° Mag. 3117”; Frosinone Province, Pastena Municipality, Pastena Caves, outside, [41.49692° N 13.48976° E; uncertainty = 150 m], [195–215 m a.s.l.], 8.VI.1986, E. Migliaccio leg., 1 ♂ macr., CEM, *verbatim labels:* “Lazio (FR) | Grotte di Pastena | esterno 8.VI.86 | leg. E. Migliaccio || ex collezione | Migliaccio | n° Mag. 3117”.

**Remarks.** Stefani [10] (p. 84) described *Embia tyrrhenica* based on specimens from southern Sardinia and mainland Italy (Rome), both macropterous, micropterous, and apterous.

A few years earlier, Silvestri [27] had identified some winged male specimens from the area near Rome, such as *E. savignyi* Westwood, 1837, a species described as being from Sudan. This author hypothesizes that they come from an introduction [27].

In fact, the specimens we examined from Central Italy (Latium and Tuscany), all macropterous, show that the 10 LP acuminate and abruptly curve outward at 90° before the apex (Figure 1a,d,g,j). This matches well with what was probably observed by [27]. LPPT P is narrow and claw-like (Figure 3a–h), actually very similar to *E. savignyi*, as noted by Silvestri [27]. The LC_1_ of the specimens we examined has a pronounced curvature, starting abruptly near the half of the basal segment of the left cercus.

However, *E. savignyi* is a very geographically distant species, with morphological characteristics also very different from the specimens from continental Italy, with a golden head, while the studied specimens have, for example, a dark brown head. Therefore, we definitively exclude this species from the Italian fauna, as also noted by Fontana et al. [9].

**Differential diagnosis.** *Embia tyrrhenica* differs from *E. pandateriensis* sp. nov. (from Ventotene island, see below) for the LC_1_ is robust and with a significant curvature, abruptly occurring near the half (Figure 4a–d) vs. elongated and slender, pronounced and located below the half of the segment (Figure 4e), and the 10 LP unfolded (Figure 1c,f,i,l) vs. folded inward (Figure 1o).

**Figure 2:**
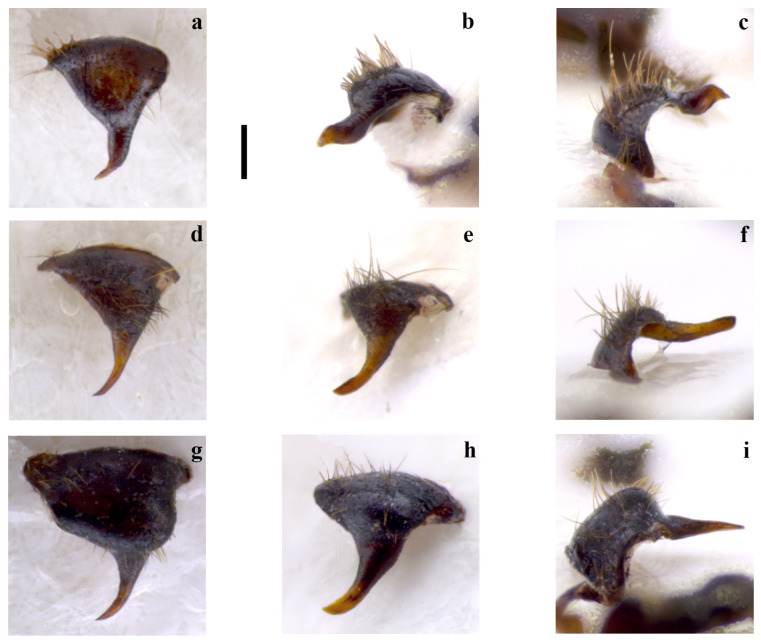
Left hemitergite of the tenth segment (10 L) in the dorsal (**a**,**d**,**g**), process of the 10 L (10 LP) from below (**b**,**e**,**h**) and from the inner side (**c**,**f**,**i**) of *Embia ramburi* Rimsky-Korsakow, 1905 (**a**–**c**), *E. specolensis* sp. nov. (**d**–**f**), and *E. brutia* sp. nov. (**g**–**i**). Scale bar = 0.2 mm. Photos by Fabio Cianferoni.

**Figure 3:**
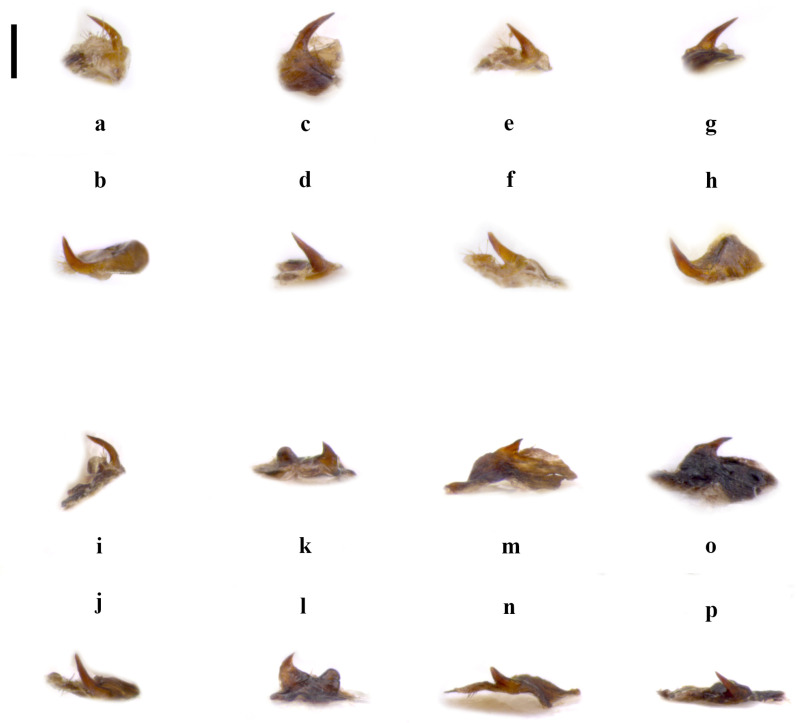
Process of the left paraproct (LPPT P) from two different sides of *Embia tyrrhenica* Stefani, 1953 (**a**–**h**), *E. pandateriensis* sp. nov. (**i**,**j**), *E. ramburi* Rimsky-Korsakow, 1905 (**k**,**l**), *E. specolensis* sp. nov. (**m**,**n**), and *E. brutia* sp. nov. (**o**,**p**). Scale bar = 0.2 mm. Photos by Fabio Cianferoni.

**Figure 4:**
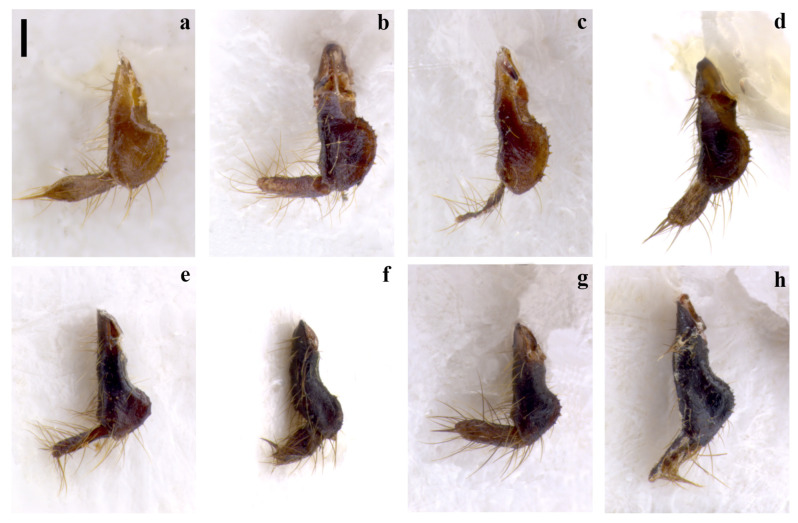
Left cercus (LC_1_ + LC_2_) in the dorsal view of *Embia tyrrhenica* Stefani, 1953 (**a**–**h**), *E. panda-teriensis* sp. nov. (i,j), *E. ramburi* Rimsky-Korsakow, 1905 (k,l), *E. specolensis* sp. nov. (m,n), and *E. brutia* sp. nov. (o,p). Scale bar = 0.2 mm. Photos by Fabio Cianferoni.

It can be separated from *E. nuragica* (so far known only from Sardinia) and *E. brutia* sp. nov. (from Calabria, see below) by LC1 with curvature starting from its half (Figure 4a–d) vs. curvature, with the widest part located widely below the lower half [10] (p. 89, Figure 2b), [19] (p. 301, Figure 11), Figure 4h; the 10 LP (in the dorsal view), which narrows into a tip in the distal part (Figure 1a,d,g,j) vs. with a more robust appearance that maintains the same width almost up to the distal part [10] (p. 89, Figure 2b), [19] (p. 301, Figure 11) (Figure 2g), the LPPT P claw-like (Figure 3a–h) vs. thorn-shaped [10] (p. 89, Figure 2d), [19] (p. 301, Figure 11), (Figure 3o,p).

*Embia tyrrhenica* can be immediately separated from *E. ramburi* and *E. specolensis* sp. nov. (see below) based on the morphology of the 10 LP—especially from the inner and external side—(Figure 1b,c,e,f,h,i,k,l vs. Figure 2b,c,e,f), the LPPT P claw-like (Figure 3a–h) vs. thorn-shaped (Figure 3k,l,m,n), and the LC_1_ strongly truncated in *E. ramburi* (Figure 4f).

*Embia tyrrhenica* can be distinguished from *E. minapalumboi* (from Madonie, Sicily) mainly due to the shape of the hypandrium (H) and its process (cf. Figure 6a), i.e., sickle-shaped with the very long process in the second species (cf. Figure 6b).

It can be separated from *E. girolamii* (known only from a site on the northern coast of Tuscany) due to the 10 LP being very robust and truncated in the latter, the very peculiar morphology LPPT P, and LC_1_, which progressively widens basally without a clear break [24] (p. 49, Figure 6); from *E. cynthiae* (known only from north-eastern Sardinia) mainly due to the morphology of the 10 LP, robust and triangular, ending in a small tip, the LPPT P very short and not claw-like, and the basal segment of the left cercus without a basal curvature starting abruptly [25] (p. 45, Figure 3).

**Distribution.** The species is recorded from mainland Italy and the islands and from Croatia (see, e.g., [9]), but the literature records, e.g., [4], require confirmation after a three-dimensional examination of the male terminalia. In addition to Sardinia (type locality), the occurrence in Tuscany and Latium is confirmed after these records.

***Embia* cf. *tyrrhenica* Stefani, 1953**

**Material examined.** Tuscany: Florence Metropolitan City, Sesto Fiorentino Municipality, National Research Council of Italy, Florence headquarters, [43.818972° N 11.200792° E; uncertainty = 30 m], [39 m a.s.l.], under a bat colony (*Pipistrellus kuhlii*), V.2023, L. Ancillotto leg., 1 ♀ apt., 1 immature (macr.), CNRF, *verbatim labels:* “Sesto Fiorentino, sede CNR-IRET, sotto colonia *P. kuhlii*, maggio 2023, Leonardo Ancillotto”.

**Remark.** An isolated female and an immature wing pad (probably future macropterous). Very likely they belong to *E. tyrrhenica* since, according to the collector, they were found a week apart at the same site.

**Figure 5:**
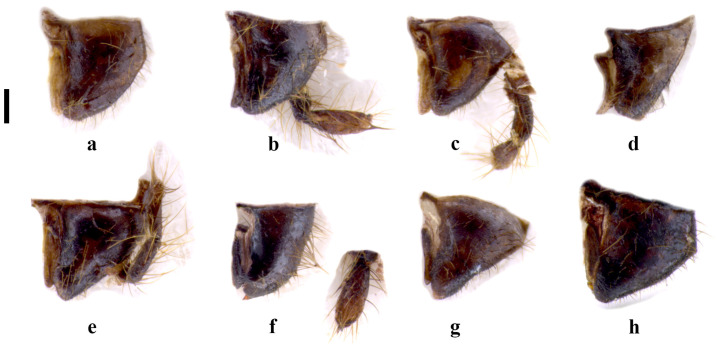
Right hemitergite of the tenth segment (10 R) of *Embia tyrrhenica* Stefani, 1953 (**a**–**d**), *E. pandateriensis* sp. nov. (**e**), *E. ramburi* Rimsky-Korsakow, 1905 (**f**), *E. specolensis* sp. nov. (**g**), and *E. brutia* sp. nov. (**h**). Scale bar = 0.2 mm. Photos by Fabio Cianferoni.

***Embia pandateriensis*** **sp. nov.**
  (Figures 1m–o, 3i,j, 4e, 5e, 7b and 9m,n)
  urn:lsid:zoobank.org:act:09701698-8F57-4444-9062-4F7297922A71

*Embia tyrrhenica* Stefani, 1953. Fontana [25] (p. 42), *partim* (“micropterous male” of Ponza island)—possible misidentification.

**Diagnosis.** Medium-sized dark *Embia* with appendages and head brown–reddish; male (holotype brachypterous) is characterized by 10 LP (in dorsal view) sharpened in the distal part, very curved, unfolded (seen from inner side), LPPT P claw-like, and LC_1_ with pronounced curvature, starting gently well below its half.

**Derivatio nominis.** The species is named after the name Pandateria (from Παντατήρια [Pantateria] in Ancient Greek), which Romans used to call the island of Ventotene, the place of origin of the known specimen; adjective.

**Material examined.** HOLOTYPE (brachypterous male; Figure 7b). Lazio: Latina Province, Ventotene Municipality, Ventotene Island, surroundings north-west of Cala Rossano, [40.80000° N 13.42945° E; uncertainty = 250 m], [15–45 m a.s.l.], 4.VII.1995, M.F. Zampetti leg., CEM, coll. n. 23304, *verbatim labels:* “Ventene [sic!] dint. NO | Cala Rossa [sic] | 4.VII.95 | M. F. Zampetti || ex collezione | Migliaccio | n° Mag. 3117 || Holotypus | *Embia pandateriensis* sp. nov. ♂ | Cianferoni & Ceccolini, 2024”.

**Description of the holotype (male).** Appearance: Medium-sized, brachypterous; dark brown except the legs, antennae, and some anterior parts of head brown–reddish; with numerous hairs all over the body (appendages included), shorter in the antennae and wings and different in length in the head, thorax, abdomen, and legs.

Cranium sub-quadrate with straight sides, scarcely elongate, devoid of dorsal pattern.

Antennae (incomplete) brown–reddish, almost unicolorous. First antennal segment clearly larger than the following; third antennal segment about 1.5 times (more in the right antenna) longer than the second; fourth antennal segment subequal in length to the second but wider; the fifth antennal segment shorter in length than the third; from the sixth antennal segments progressively longer and thinner.

Eyes small, slightly projecting.

Clypeus with anterior margin slightly emarginated and with two posterolateral ferruginous spots.

**Figure 6:**
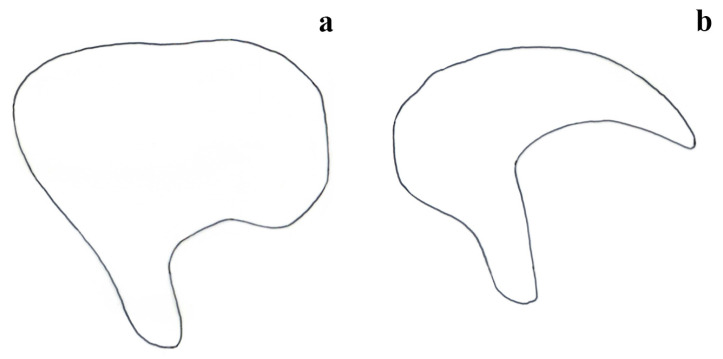
Comparison between two types of hypandrium (H). Subquadrate H, most of the *Embia* spp. (**a**); and sickle-shaped H, *E. minapalumboi* Fontana, 2024 (**b**). Original drawings by F. Cianferoni.

Terminalia: 10 L slightly enlarged; 10 LP in the dorsal view sharpened in the distal part and very curved (Figure 1m) in the inner view, which progressively becomes thinner, unfolded inward and with apex slightly tapered (Figure 1o); 10 R triangular (Figure 5e); MF not very sclerotized; LPPT P well developed, narrow and claw-like (Figure 3i,j). LC_1_ with pronounced curvature, starting gently well below its half (Figure 4e); H enlarged, subquadrate (cf. Figure 6a).

Dimensions: body length 7.8 mm; cranium length 1.53 mm, width 1.18 mm; fore wing length 2.15 mm, width 0.70 mm (hind wings are similar in size, but an accurate measurement was avoided so as not to damage the only known specimen already mounted); LC_1_ length 0.73 mm, width 0.26 mm.

**Remarks.** This insular brachypterous specimen is quite similar to *E. tyrrhenica*, in which all the specimens we examined are macropterous.

The male terminalia shows marked differences. The 10 LP is similar in shape but not folded inward (Figure 1o); it looks more robust in the dorsal view, and its apex is less tapered, but this is actually due to the different orientation (cf. Figure 1). The well-developed LPPT P (Figure 3i,j), narrow and claw-like, is similar to that of *E. tyrrhenica* (cf. Figure 3). The basal segment of the left cercus (Figure 4) is probably the best diagnostic characteristic for separation since it is more slender compared to that of *E. tyrrhenica*. Its lobe curvature is pronounced and starts gently well below the half of LC_1_ (while the lobe curvature of *E. tyrrhenica* is more regular, and the lobe begins abruptly at the half of the basal segment of the left cercus). Echinulations are blunt and not obvious.

The characteristics are sufficiently evident to separate this species from the previous one, which presents constant characteristics in all the specimens examined, which come from different localities. At the same time, the affinities with the previous species suggest a probable phylogenetic closeness, which can only be verified by further investigations (molecular and morphological).

It is interesting to note that Ross [18] hypothesized that close studies could lead to “distinguishable populations, or even a complex of races or weak species” in *E. tyrrhenica*.

**Differential diagnosis.** This species can be separated from all the other known species of *Embia* occurring in Italy (except *E. tyrrhenica* and *E. minapalumboi*) by the 10 LP, seen in the dorsal view, with a strong curvature between 45 and 90 degrees. It is similar to *E. tyrrhenica* but easily separable from the latter by the LC_1_, slender, with a pronounced lobe located below the half of the segment (Figure 4e) vs. lobe curvature more rounded and abruptly occurring near the half of the segment (Figure 4a–d), and for the 10 LP, seen in the inner view, straight (Figure 1o) vs. more or less evidently but always folded inward (Figure 1c,f,i,l). *Embia minapalumboi* also has a different lobe curvature of the LC_1_, more rounded and occurring abruptly (similar to *E. tyrrhenica*), as well as a slightly different curvature of the 10 LP in the dorsal view, different shape of the 10 R in the ventral view (with a peculiar shape of hypandrium, i.e., sickle-shaped with the very long process) and less developed LPPT P (cf. [21]).

**Distribution.** To date, known only for the island of Ventotene. Probably, it can also be found on other Pontine islands (see above).

**Figure 7:**
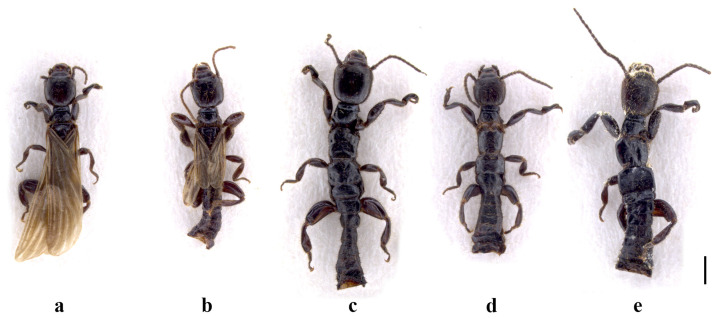
Male dorsal habitus of *Embia tyrrhenica* Stefani, 1953 (**a**), *E. pandateriensis* sp. nov. (**b**), *Embia ramburi* Rimsky-Korsakow, 1905 (**c**), *E. specolensis* sp. nov. (**d**), and *E. brutia* sp. nov. (**e**). Terminalia removed. Scale bar = 0.2 mm. Photos by Fabio Cianferoni.

***Embia ramburi*** **Rimsky-Korsakow, 1905**
  (Figures 2a–c, 3k,l, 4f, 5f, 6a, 7c and 9o,p)

*Embia ramburi* Rimsky-Korsakow, 1905. Fontana et al. [32] (p. 85, 88).

**Diagnosis.** Medium-sized dark *Embia*, with males (apterous) characterized by 10 LP with very robust basal part and a thinner distal part, peculiarly twisted when observed in the inner view, LPPT P tooth-like, associated with a large dome-like nodule, and LC_1_ sinuated, with a gentle curvature, slightly pronounced in the basal part, cut sharply where the left cercus is connected.

**Material examined.** Tuscany: Florence Metropolitan City, Fiesole Municipality, Caldine, 43.836915° N 11.309579° E (uncertainty = 2 m), 201 m a.s.l., vagrant on a terracotta step, 3.V.2024, F. Graziani and F. Cianferoni leg., 1 ♂ apt., CFG, *verbatim labels:* “Toscana: Fiesole (Firenze), Caldine, 43,836915°N 11,309579°E (incertezza = 2 m), 201 m s.l.m., vagante su uno scalino in cotto, 3.V.2024, F. Graziani & F. Cianferoni leg.”; Grosseto Province, Isola del Giglio Municipality, Giglio Island, [42.35348° N 10.90161° E, un = 5 km], [0–400 m a.s.l.], 11.V.1969, E. Migliaccio leg., 1 ♂ apt., CEM, *verbatim labels:* “Isola del Giglio | 11.V.69 | leg. E. Migliaccio || Embia | ramburi || ex collezione | Migliaccio | n° Mag. 3117”; Lazio: Rome Capital Metropolitan City, service station on the A24 motorway Colle Tasso Nord, [41.92749° N 2.73899° E; un. = 200 m], [ca. 50 m a.s.l.], 3.VI.2005, M.F. Zampetti leg., 1 ♂ apt. (Figure 7c), CEM, *verbatim labels:* “Roma, Stazione | di servizio su A24 | Colle Tasso Nord | 3.6.05-M.F.Zampetti || ex collezione | Migliaccio | n° Mag. 3117”.

**Remarks.** This apterous specimen shows very peculiar features, appreciable only in a three-dimensional study (see Materials and Methods and Discussion for further information). It has the 10 LP, in the dorsal view (Figure 2a), with a very robust basal and thinner distal parts. As already mentioned, however, only by observing this piece from different angles, i.e., from the inner and external sides (Figure 2c,b), can its real morphology be appreciated. In fact, the distal part tapers into a twisted plate with a tapered tip. From certain angles, it resembles the head of a passerine bird. This characteristic alone immediately allows us to separate this species from all the other congeneric species.

LPPT (Figure 3k,l) has a large dome-like nodule; LPPT P is a robust and short tooth-like hooked denticle, different in shape and length from the other related species. This characteristic is also good for separation.

LC_1_ (Figure 4f) is elongated and sinuated, with a gentle curvature, slightly pronounced in the basal part. The segment is cut sharply where the left cercus is connected.

These characteristics match quite well with the specimens from Marche reported by Fontana et al. [32] and identified as *E. ramburi*. Unfortunately, it is impossible to compare the 10 LP from different views. The slide mounting is limiting in this case (see also Discussion).

*Embia ramburi* was described from the Nice area, in southern France. The type was not located, but a redescription based on a topotypic male was provided by Ross (1966). The specimens reported by Fontana et al. [32] and identified as *E. ramburi* seem different from each other and even the one from the south of France (near Montpellier) does not match well with that of Ross’ [18] redescription of *E. ramburi*. However, 10 LP is difficult to compare in only the dorsal view, see, e.g., also [9].

**Differential diagnosis.** This species can be immediately separated from all the other *Embia* occurring in Italy by the shape of its 10 LP. In particular, from an inner and external view, it is easy to appreciate the peculiarly twisted morphology of the process of the 10 L, which distinguishes this species from others. Even the LC_1_ is sharply cut at the point where the cercus is connected. This characteristic is also unique compared to other congeneric species.

**Distribution.** In addition to the type locality (southern France), it is recorded from mainland, insular Italy, and Spain, see, e.g., [9,31], but the precise distribution will need to be ascertained based on the three-dimensional examination of the male terminalia. All the previous literature records require confirmation. It is now confirmed for Tuscany and Lazio.

***Embia specolensis* sp. nov.**
  (Figures 2d–f, 3m,n, 4g, 5g, 7d and 9q,r)
  urn:lsid:zoobank.org:act:1B21729F-DD21-4C45-85FD-3A7F3BC2FCEA

**Diagnosis.** Medium-sized dark *Embia*, with the male (holotype apterous) characterized by the 10 LP sharpened in the distal part and slightly curved, and (in the inner view) knife-shaped and subrectangular, LPPT tooth-like, and LC1 with lobe starting clearly below the median part and very pronounced

**Derivatio nominis.** The species refers to “La Specola”. The name is used to indicate a section of the Natural History Museum of Florence in which the known specimens were collected; adjective.

**Material examined.** HOLOTYPE (apterous male; Figure 7d). Tuscany: Florence Metropolitan City, Florence Municipality, Florence, “La Specola” (Natural History Museum), museum garden, 43.76390° N 11.24788° E (uncertainty = 15 m), 75 m a.s.l., 30.V.2016, F. Ceccolini and L. Pizzocaro leg., CFCe, *verbatim labels:* “Toscana: Firenze (Giardino | “La Specola”), 75 m | 11°14′ E/43°45′ N—30.V.2016 | Leg. F. Ceccolini & L. Pizzocaro || Coll. Ceccolini | 37 || Holotypus | *Embia specolensis* sp. nov. ♂ | Cianferoni & Ceccolini, 2024”. PARATYPES. Tuscany: Florence Metropolitan City, Florence Municipality, Florence, “La Specola” (Natural History Museum), museum garden, silk tubes under a wooden plank, 43.763838° N 11.247603° E (uncertainty = 1 m), 62 m a.s.l., 19.X.2023, F. Ceccolini and F. Cianferoni leg., 1 immature specimen (pure ethanol), CFCi; *verbatim labels:* “Toscana: Firenze, “La Specola”, giardino, gallerie di seta sotto un’asse di legno, 43,763838° N 11,247603° E (incertezza = 1 m), 62 s.l.m., 19.X.2023, F. Ceccolini & F. Cianferoni leg. || Paratypus | *Embia specolensis* sp. nov. ♂ | Cianferoni & Ceccolini, 2024”; *idem*, reared by F. Cianferoni and collected on 29.V.2024, 1 ♂, 2 ♀♀ apt. + 1 ♂, 1 ♀ apt. (pure ethanol), CFCi, idem1 ♀ apt., CFCe; 1 ♀ apt., MZUF, coll. n. 23305; 1 ♀ apt., CNRF, *verbatim labels:* “Toscana: Firenze, “La Specola”, giardino, gallerie di seta sotto un’asse di legno, 43,763838° N 11,247603° E (incertezza = 1 m), 62 s.l.m., 19.X.2023, F. Ceccolini & F. Cianferoni leg., ex allevamento F. Cianferoni leg. 29.V.2024 || Paratypus | *Embia specolensis* sp. nov. ♂/♀ | Cianferoni & Ceccolini, 2024”.

Further non-type material: *idem*, reared by F. Cianferoni and collected on 29.V.2024, one immature specimen died prematurely (pure ethanol), CFCi.

**Description of the holotype (male).** Appearance: medium-sized, apterous; dark brown; with numerous hairs all over the body (appendages included), shorter in the antennae and different in length in the head, thorax, abdomen, and legs.

Cranium sub-quadrate with slightly rounded, scarcely elongated, devoid of dorsal pattern.

Antennae (only the right, incomplete) brown, almost unicolorous. First antennal segment clearly larger than the following; third antennal segment 1.5 times longer than the second; fourth antennal segment subequal in length to the second but distinctly wider; the fifth antennal segment subequal in length to the third but larger; from the sixth antennal segment progressively longer and thinner.

Eyes small, not projecting.

Anterior margin of the clypeus slightly emarginated. Right mandible with two teeth, left mandible hidden (three teeth in that of the paratype).

Legs brown.

Terminalia: 10 L not enlarged; 10 LP in the dorsal view sharpened in the distal part and slightly curved (Figure 2d,e), in the inner view, knife-shaped and of constant width (Figure 2f); 10 R triangular (Figure 5g); MF not very sclerotized; LPPT P small, with a broad base and tooth-like (Figure 3m,n). LC_1_ with lobe starting clearly below the median part and very pronounced (Figure 4g); H enlarged, subquadrate (cf. Figure 6a).

Dimensions (in brackets, paratype measurements; the second paratype, longer, is preserved in ethanol): body length 8.1 (8.7, 9.2) mm; cranium length 1.38 (1.60, 1.50) mm, width 1.15 (1.27, 1.20) mm; LC_1_ length 0.73 (0.73, 0.75) mm, width 0.25 (0.26, 0.30) mm.

Female. Similar to the male, but more robust and larger. Slightly lighter in color (red–dish brown); dorsal pattern with reddish stripes restricted to the posterior half of the head, evident in some specimens. Long golden hairs on the head (darker in males).

Dimensions (ranges of paratype measurements): body length 10.4–12.9 mm; cranium length 1.75–2.05 mm, width 1.40–1.70 mm.

**Remarks.** This apterous specimen belongs to an undescribed species, which could have been confused with *E. ramburi* Rimsky-Korsakow, 1905 until now. According to Ross (1966: 297), the repository of the type of the latter species is unknown, and it may not exist; its redescription and figuring [18] are based on a topotypic male (from southern France) belonging to a series provided by Renzo Stefani.

However, *E. ramburi* shows a 10 LP less sharpened in the distal part and blunt in the dorsal view. At the same time, the 10 LP new species is, in the dorsal view, much thinner and pointed terminally (see Differential diagnosis below). The LC_1_ is similar to that of the redescription by Ross [18], but the new species presents a more sudden widening of the basal part (while it is more constant in *E. ramburi*). The enlarged basal part has a more pronounced curvature than the gentler one of *E. ramburi*, cf. [10] (p. 91, Figure 3d).

**Differential diagnosis.** This species can be separated from the most similar species: from *E. ramburi* (type locality in southern France) by the 10 LP (in the dorsal view) sharpened in the distal part (Figure 2d) vs. robust and blunt [10] (p. 91, Figure 3d), 18 (p. 298, Figure 9), (Figure 2a); from *E. brutia* sp. nov. (see below) by the 10 LP (in the inner view) knife-shaped and of constant width (Figure 2f) vs. decreasing in width distally and terminating in a tip, since it is actually slightly twisted (Figure 2i); from *E. nuragica* by the 10 LP (in the dorsal view) thin and curved (Figure 2d) vs. robust (knife-shaped and of constant width) and straight [10] (p. 89, Figure 2b), [18] (p. 301, Figure 11); from *E. ilvana* mainly for the different shape of 10 LP and for the mandibles bear a robust dorsal-subapical tooth, cf. [22].

**Distribution.** Currently known only from the type locality (Central Italy, Florence).

***Embia brutia*** **sp. nov.**
  (Figures 2g–i, 3o,p, 4h, 5h, 7e and 9a,b)
  urn:lsid:zoobank.org:act:410822E7-7451-4150-925C-8DB616D41B03

**Diagnosis.** Medium-sized dark *Embia*, with males characterized by 10 LP robust, knife-shaped and slightly curved, in the inner view it appears to be decreasing in width distally and terminating in a tip (since it is actually slightly twisted), LPPT small and tooth-like, and LC_1_ with lobe starting clearly below the median part and very pronounced.

**Derivatio nominis.** The specific epithet is an adjective derived from the Bruttians (Bruttii in Latin), an ancient Italic people inhabiting the southern extremity of Italy, roughly corresponding to the modern region of Calabria. The pronunciation, using the International Phonetic Alphabet (IPA), is “bruːtsjä”.

**Material examined.** HOLOTYPE (apterous male; Figure 7e). Calabria: Vibo Valentia Province, Dinami Municipality, Marepotamo River, [38.54954° N 16.12394° E; uncertainty = 50 m], [82–88 m a.s.l.], flood debris, 25.IV.1994, S. Cianfanelli, E. Talenti leg., MZUF, coll. n. 23306, *verbatim labels:* “Calabria: Fiume Marepotano [sic] (Dinami: | CZ), posatura | 25/IV1994 S. Cianfanelli & E. Talenti! || Holotypus | *Embia brutia* sp. nov. ♂ | Cianferoni & Ceccolini, 2024”.

**Description of the holotype (male).** Appearance: medium-sized, apterous; dark brown; with numerous hairs all over the body (appendages included), shorter in the antennae and different in length in the head, thorax, abdomen, and legs.

Cranium sub-quadrate slightly elongate; dorsal pattern with a reddish median stripe restricted to the posterior half of the head, two smaller reddish sublateral stripes near the posterior margin, and lateral sides of the head reddish.

Antennae (incomplete) brown, almost unicolorous. First antennal segment clearly larger than the following; third antennal segment more than two times longer than the second in the right antenna, 1.5 times longer than the second in the left one; fourth antennal segment subequal in length to the second but slightly wider; from the sixth antennal segments progressively longer.

Eyes small, not projecting.

Anterior margin of clypeus slightly emarginated. Right mandible with two teeth, left mandible with three teeth.

Legs brown.

Terminalia: 10 L very enlarged; 10 LP robust, knife-shaped, and slightly curved (Figure 2g,h), in the inner view, it appears to be decreasing in width distally and terminating in a tip, since it is actually slightly twisted (Figure 2i); 10 R subtriangular (Figure 5h); MF not very sclerotized; LPPT P quite small, with a broad base and moderate elongated tooth-like (Figure 3o,p); LC_1_ with lobe starting clearly below the median part and very pronounced (Figure 4h); H enlarged, subquadrate (cf. Figure 6a).

Dimensions: body length 9.7 mm; cranium length 1.80 mm, width 1.40mm; LC_1_ length 0.80 mm, width 0.33 mm.

Female. Unknown.

**Remarks.** This apterous specimen collected in Calabria (Southern Italy) has an affinity with *E. ramburi* (obviously, at this stage, talking about “groups of species” still makes no sense since an objective analysis, such as a molecular phylogeny, is still missing). However, it can be immediately separated from *E. ramburi* (whose type locality, southern France, is very distant geographically) due to the very different morphology of the terminalia.

Apparently, the most similar species is *E. nuragica* (only known from Sardinia). The LC_1_ and LPPT P match quite well in the two species; however, the 10 LP of *E. nuragica* shows a rather straight shape in the dorsal view [10] (p. 89, Figure 2), [18] (p. 301, Figure 11)], while that of the new species from Calabria has a strong and evident curvature in the distal part (Figure 2g).

There is also an affinity with *E. specolensis* sp. nov., described above, but the morphology of the 10 LP (observed in different positions) allows a clear separation. Also, the shape of the LPPT P and LC_1_ has some differences (see below in the Differential diagnosis for details).

**Differential diagnosis.** This species can be separated from the most similar species by the following features: from *E. ramburi* (type locality in southern France) by the 10 LP (in the dorsal view) sharpened in the distal part (Figure 2g) vs. blunt [10] (p. 90, Figure 3d), [18] (p. 298, Figure 9)]; from *E. specolensis* sp. nov. by the 10 LP (in the inner view) appears to be decreasing in width distally and terminating in a tip, since it is actually slightly twisted (Figure 2i) vs. knife-shaped and of constant width (Figure 2f); from *E. nuragica* by the 10 LP (in the dorsal view) slightly but curved (Figure 2g) vs. straight [10] (p. 89, Figure 2b), [18] (p. 301, Figure 11)]; from *E. ilvana* mainly by the different shape of 10 LP and for the mandibles bear a robust dorsal-subapical tooth, cf. [22].

**Distribution.** Known only from the type locality in southern Calabria.

***Embia* sp. 1**

**Material examined.** Sardinia: Nuoro Province, Sorgono Municipality, Sorgono, [40.02851° N 9.10378° E; uncertainty = 1000 m], [650–800 m a.s.l.], 2013, A. Krausse leg., 1 apterous female, 1 immature, MZUF, *verbatim labels:* “Sardegna | Sorgono | A. Krausse, 1913 || *Embia* sp. ♀| Det. F. Cianferoni, 2024”.

**Remark.** Isolated female not possible to identify at the species level.

***Embia* sp. 2**

**Material examined.** Tuscany: Livorno Province, La Scola Islet near Pianosa Island, [42.58378° N 10.10640° E; uncertainty = 100 m], [0–30 m a.s.l.], 11.VI.1998, L. Bartolozzi, B. Cecchi, L. Dapporto, P. Lo Cascio and A. Sforzi leg., 1 apterous female (from immature specimen), MZUF, *verbatim labels:* “Toscana: Isola di Pianosa (LI), | Isolotto La Scola 11.VI.1998 | ex larva legit L. Bartolozzi B. | Cecchi, L. Dapporto, P. Lo Cascio | & A. Sforzi (Num. Mag. 2100) || *Embia* sp. ♀ | Det. F. Cianferoni, 2024”.

**Remark.** Isolated female not possible to identify at the species level. However, this is the first record of the order Embioptera for La Scola Islet, Tuscan Archipelago, cf. [22].

***Embia* sp. 3**

**Material examined.** Lazio: Rome Capital Metropolitan City, ancient Appian Way (road), [41.81857° N 12.55849° E; uncertainty = 1500 m], 100 m a.s.l., 13.II.2001, M.F. Zampetti leg., in soil with lichen (collected 1.III.2001), 1 apterous female, CEM, *verbatim labels:* “Roma, Appia antica | m 100, 13.02.2001 | M.F.Zampetti || in terreno con lichene | raccolto il 01.03.2001 | [embiottero] || ex collezione | Migliaccio | n° Mag. 3117 || *Embia* sp. ♀ | Det. F. Cianferoni, 2024”.

**Remark.** Isolated female not possible to identify at the species level.

***Embia* sp. 4**

**Material examined.** Tuscany: Arezzo Province and Municipality, Arezzo, Via Masaccio, 43.45977° N 11.87405° E; uncertainty = 10 m, 255 m a.s.l., 12.VII.2019 F. Ceccolini leg., 1 apterous female, CFCe, *verbatim labels:* “Arezzo (Via Masaccio) | 43,45977° N 11,87405° E (inc. 10 m) | 255 m, 12.VII.2019 F. Ceccolini! || *Embia* sp. ♀ | Det. F. Cianferoni, 2024””.

**Remark.** Isolated female not possible to identify at the species level.

**Family Oligotomidae Enderlein, 1909**
**Genus *Haploembia* Verhoeff, 1904**

**Diagnosis.** Adult and immature specimens with two ventral papillae on the hind basitarsus. Males always apterous. Basal segment of the left cercus (LC_1_) enlarged but not developing into a distinct lobe; its surface without echinulations.

***Haploembia solieri* (Rambur, 1842)**

**Material examined.** Tuscany: Livorno Province, Elba Island, Portoferraio Municipality, Portoferraio, Forte Inglese [= English Fort], [42.81672° N 10.31835° E; uncertainty = 60 m], [35 m a.s.l.], 24.IV.2023, G. Innocenti leg., 1 immature, MZUF (ethanol collection), *verbatim labels:* “Mag. N° 1828 | Toscana: LI, Isola d’Elba | Portoferraio, Forte Inglese | 24.IV.2023 G. Innocenti! || *Haploembia solieri* | Rambur, 1842 | Det. F. Cianferoni, 2024”; Livorno Province, Pianosa Island, Golfo della [gulf of the] Botte, [42.58651° N 10.06287° E; uncertainty = 700 m], [0–30 m a.s.l.], 30.III–01.IV.1998, L. Bartolozzi, L. Dapporto and A. Sforzi leg., 1 immature, MZUF, *verbatim labels:* “Toscana: Isola di Pianosa (LI), | golfo della Botte 30.III-01.IV.1998, | legit L. Bartolozzi, L. Dapporto, | A. Sforzi (num. Mag. 2100) || *Haploembia solieri* | Rambur, 1842 | Det. F. Cianferoni, 2024”. Grosseto Province and Municipality, Poggio di [=hill of] Moscona, [42.81454° N 11.15303° E; uncertainty = 1000 m], [100–316 m a.s.l.], IV.1943, A. Andreini leg., 1 immature, MZUF, *verbatim labels:* “Moscona | (dint. Grosseto) | IV-43 | Dr. A. Andreini || *Haploembia solieri* | Rambur, 1842 | Det. F. Cianferoni, 2024”; Grosseto Province, Orbetello Municipality, Agriturismo “La Valentina”, 11.1389° E 42.5908° N; uncertainty = 500 m, 45 m a.s.l., 28.V.2002, 1 immature, F. Ceccolini leg, CFCe, *verbatim labels:* “Toscana: Agriturismo “La | Valentina”, Orbetello (GR), | 11,1389° E 42,5908° N (WGS83; | inc. 500 m), 45 m—28.V.2002 || *Haploembia solieri* | Rambur, 1842 | Det. F. Ceccolini, 2016 || Coll. Ceccolini 38”; *idem*, 42.58967° N 11.13889° E; [uncertainty = 50 m], ca. 50 m a.s.l., 25.V.2016 F. Ceccolini, F. Cianferoni and G. Mazza leg., 1 apterous female, CFCe, *verbatim labels:* “Toscana: Agriturismo “La | Valentina”, Orbetello (GR), | 11°08′20′′ E 42°35′23′′ N, ~ 50 m | 25.V.2016—F. Ceccolini, F. | Cianferoni and G. Mazza leg. || *Haploembia solieri* | Rambur, 1842 | Det. F. Ceccolini, 2016 || Coll. Ceccolini 39”; Grosseto Province, Isola del Giglio Municipality, Giglio Island, 42.318° N 10.919° E; uncertainty = 100 m, 15–30 m a.s.l., 1.V.2023, M. Morbidelli and E. Tricarico leg. (soil sample), extracted with Berlese funnel by G. Mazza, 1 apterous male, 2 apterous females, CFCi, *verbatim labels:* “[same as before] || *Haploembia solieri* | Rambur, 1842 | Det. F. Cianferoni, 2024”; Grosseto Province, Isola del Giglio Municipality, Giannutri Island, Punta [=cape] San Francesco, [42.25417° N 11.11593° E; uncertainty = 300 m], [0–15 m a.s.l.], 9.V.1992, S. Taiti, S. Vanni, A. Sforzi, M. Bucci and G. Gruber leg., 1 immature, MZUF, *verbatim labels:* “Toscana: Is. Giannutri (GR) Punta | S. Francesco 9.V.1992 Taiti, S. Vanni, | A. Sforzi, M. Bucci, G. Gruber!(Mag.1695) || *Haploembia solieri* | Rambur, 1842 | Det. F. Cianferoni, 2024”. Apulia: Taranto Province, Martina Franca Municipality, Martina Franca, Valle d’[=valley of]Itria, [40.72312° N 17.35262° E; uncertainty = 4 km], ca. 400 m a.s.l., 30.VII.2010, V. Valentini leg., 1 apterous female, CEM, *verbatim labels:* “Puglia (TA) | Martina Franca 400 m | Valle d’Itria 30/VII/2010 | leg. V. Valentini || ex collezione | Migliaccio | n° Mag. 3117|| *Haploembia solieri* | Rambur, 1842 | Det. F. Cianferoni, 2024”. Sardinia: South Sardinia Province, Arbus Municipality, 39.55310° N 8.56616° E; uncertainty = 50 m, [360 m a.s.l.], in gut content of *Speleomantes genei* (Temminck and Schlegel, 1838), collected by stomach flushing, 18.I.2024, E. Lunghi leg., 2 immatures, CFCi, *verbatim labels:* “[same as before] || *Haploembia solieri* | Rambur, 1842 | Det. F. Cianferoni, 2024”.

**Remark.** *Embia solieri* Rambur, 1842 (subsequently transferred to the genus *Haploembia*) was described from southern France (Marseille) on a small nymph (sex undeterminable) and currently, according to Ross [18], lacking its head, prothorax and all the appendages. Further species described, respectively, from Crimea, Sicily, Spain, and Morocco were (and still are) treated as synonyms [18,33].

A further species, described as *Gynenmbia tarsalis* Ross, 1940, and later moved to the genus *Haploembia*, was described on parthenogenic populations from California and Arizona.

Stefani and Contini [34] listed some morphological features in the amphigoric and parthenogenetic forms of *Haploembia*, both at the level of adult females and eggs. However, *H. tarsalis* was considered a synonym of *H. solieri*.

More recently, Hodson et al. [33] provided a molecular analysis sequencing two genes, Cytochrome c oxidase subunit 1 (COI) and Histone 3 (H3). They included several specimens from the USA, two specimens from Peninsular Italy (Pisa), and two from Sardinia (Sassari). They found that the clade of asexual individuals from the USA included the two females from Sardinia and clustered with the sexual specimens from Peninsular Italy as a possible sister group. Another separate clade includes all the sexual specimens from the USA. Thus, they proposed using *Haploembia tarsalis* (Ross, 1940) for parthenogenetic populations, whether from Sardinia, other Mediterranean Islands or California.

Some years later, Kelly et al. [35] used H3 to test their species identifications and found two separate clades in California: for sexual specimens (if males were found with females) and for parthenogenetic specimens (if solitary females were found). They attributed the two groups, respectively, to *H. solieri* and *H. tarsalis*.

Recently, Fontana and Forbicioni [22] listed further specimens of *Haploembia* from the Tuscan Archipelago and Sardinia, attributing the isolated females to *H. tarsalis*, mainly based on pigmentation as suggested by Stefani and Contini [34] for the parthenogenetic populations.

As already anticipated by Hodson et al. [33], the species-level situation in *Haploembia* may be considerably more complex than is currently recognized. We believe that this genus needs an in-depth revision before reaching conclusions and attributing the literature records to existing names. In fact, we remember that the type specimen of *H. solieri* (Rambur) is problematic. Therefore, at the moment, we do not know which populations attribute to *H. solieri* if other species of *Haploembia* under the same name will be confirmed as valid species. Moreover, several available names (currently synonyms) may be usable for any other species.

In our opinion, to date, the only conclusion we can draw is that specimens from northern Sardinia and the USA belong to the same species of *Haploembia* and that it is very likely that this same species was introduced into America from the Mediterranean. Although possible (or perhaps even probable), we do not believe it is acceptable that every parthenogenetic population from the Old World should be attributed to *H. tarsalis*. The latter name, proposed for specimens from California and Arizona, was used by Hodson et al. [33] and Kelly et al. [35] only for specimens collected in the USA. Further studies will be needed before establishing that the parthenogenetic populations of Mediterranean *Haploembia* should be attributed to a species other than the sexually reproducing one.

Although there seems to be evidence that there may be more than one species under the current *H. solieri* in the Mediterranean, the most conservative solution appears to be to continue using the name *H. solieri* for them (with the obvious exception of *H. palaui* Stefani, 1955, which is well distinguishable morphologically), pending further research.

Among the materials examined, there is the first record of *Haploembia* for the Island of Giannutri, Tuscan Archipelago, cf. [22], which we consider *H. solieri*, waiting for further insights into the genus. Another two immature specimens of *H. solieri* (see previous comment) were collected by stomach flushing from the cave salamander *Speleomantes genei* and they represent a new prey record for this species, see [36] for further information.

### 3.2. Provisional Checklist of the Embioptera in Italy

Concerning *Embia* only, the verified distribution is listed (Table 1). *Haploembia* is reported as a simplified distribution as currently reported in the literature, e.g., [18,22,37].

### 3.3. Provisional Key for Adult Males of Embioptera Occurring in Italy

This key (Table 2) is partly modified after Ross [18] and Fontana et al. [9], and it is only valid for adult males, except when indicated (first point). It is a tentative key, pending further research, based on the characteristics of the male terminalia observed in the dorsal or ventral view, the only features available for all the known species, allowing a comprehensive comparison. Further research will be essential to examine the various parts three-dimensionally and possibly refine and rearrange a new key.

**Table 2:** The provisional key for adult males of Embioptera occurring in Italy.

1	Hind basitarsus (of all instars) with two ventral papillae (Figure 8b). Males always apterous .......................................................................................................... ***Haploembia***, one species (see remark above): ***H. solieri*** (Rambur, 1842) (Figure 9w,x) (described from the southern coast of France, Marseille; several records from the northern Mediterranean coasts and Tyrrhenian islands; see checklist for further distribution records)
–	Hind basitarsus (of all instars) with one ventral papilla (Figure 8c). Males apterous or alate .................................................................................................................................. 2
2 (1)	Left cercus bilobed, with an inner-basal lobe and a larger, dorso-medial distal lobe (Figure 9v). Adult males always apterous ......................................................... ***Cleomia***, one species: ***C. guareschii*** Stefani, 1953 (Figure 9u,v) (described from southern Sardinia; recorded from other central–western Mediterranean islands)
–	Left cercus with a simple inner lobe or expansion (Figure 9b,d,f,h,j,l,n,p,r,t). Adult males apterous or alate .......................................................................................... ***Embia*** 3
3 (2)	Mandibles bearing a robust dorsal-subapical tooth ................................................................... ***E. ilvana*** Fontana and Forbicioni, 2024
	Mandibles not provided with a dorsal-subapical tooth ................................................ 4
4 (3)	Process (10 LP) of the left hemitergite of the tenth segment (10 L), in the dorsal view, with apex blunt (Figure 9e) ............................................................................................... ***E. girolamii*** Fontana, 2001 (Figure 9e,f) (known so far only for a single site on the northern coast of Tuscany)
–	Process (10 LP) of left hemitergite of tenth segment (10 L), in the dorsal view, with apex more or less pointed (Figure 9a,c,g,i,k,m,o,q,s) ................................................... 5
5 (4)	10 LP, in the dorsal view, very small and tapered (Figure 9c) ............................ ***E. cynthiae*** Fontana, 2002 (Figure 9c,d) (known so far only for the holotype from northern Sardinia)
–	10 LP, in the dorsal view, well developed and discernible from 10L (Figure 9a,e,i,k,m,o,q,s) .................................................................................................................... 6
6 (5)	10 LP, in the dorsal view, with subapical constriction and apex slightly rounded (Figure 9o) ......................................................................................................................... ***E. ramburi*** Rimsky-Korsakow, 1905 (Figures 9o,p, 2a–c, 3k,l, 4f, 5f and 7c) (described from the south-eastern coast of France, east of Nice; known so far with certainty for Central Italy, Lazio)
–	10 LP, in the dorsal view, which narrows more or less evidently, but without a constriction, and sharp apex (Figure 9a,g,i,k,m,q,s) ....................................................... 7
7 (6)	H, in the ventral view, sickle-shaped. 10 LP and LC_1_ as in Figure 6b ..... ***E. minapalumboi***, Fontana, 2024
	H, in ventral view, expanded, not sickle-shaped (Figure 6a). 10 LP and LC_1_ different in shape ................................................................................................................................ 8
8 (7)	10 LP, in the dorsal view, very curved, between 45 and 90 degrees (Figure 9m,s) ............................................................................................................................................ 9
–	10 LP, in the dorsal view, straight or slightly curved, between 0 and 45 degrees (Figure 9k,a,q) ........................................................................................................................ 10
9 (8)	LC_1_ with lobe starting from the median part (Figure 9t) ........................... ***E. tyrrhenica*** Stefani, 1953 (Figures 9a,b, 1a–l, 3a–h, 4a–d, 5a–d and 7a) (known so far with certainty for Sardinia, Central Italy: Tuscany and Lazio)
–	LC_1_ with lobe starting clearly below the median part (Figure 9n) ............................................................................................................. ***E. pandateriensis*** sp. nov. (Figures 9k,l, 1m–o, 3i,j, 4e, 5e and 7b) (known so far for Ventotene Island, Pontine Islands, Lazio)
10 (8)	10 LP, in the dorsal view, straight until near its apex (Figure 9k) ............... ***E. nuragica*** Stefani, 1953 (Figure 9k,l) (known so far only for northern Sardinia)
–	10 LP, in the dorsal view, curving clearly before its apex (Figure 9c,m,q,s) ............................................................................................................................. 11
11 (10)	10 L, in the dorsal view, not enlarged, without corner (evident especially in the left side); (Figure 9q) ....................................................................................... ***E. specolensis*** sp. nov. (Figures 9q,r, 2d–f, 3m,n, 4g, 5g and 7d) (known so far with certainty only for Florence, Tuscany; holotype from the Specola garden)
–	10 L, in the dorsal view, enlarged, squared (evident especially on the left side) (Figure 9a) ...................................................................................................... ***E. brutia*** sp. nov. (Figures 9a,b, 2g–i, 3o,p, 4h, 5h and 7e) (known so far only for Southern Italy, southern Calabria)

**Figure 8:**
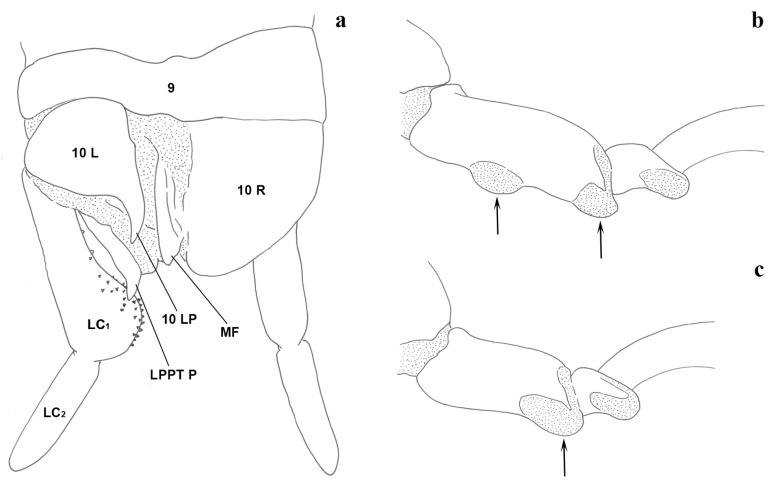
Terminalia in the dorsal view (for abbreviations, see Materials and Methods) (**a**); Hind basitarsus of *Haploembia* (**b**) and *Embia* (**c**) with the indication of ventral papillae. Redrawn with modifications from Ross (1966) by F. Cianferoni.

**Figure 9:**
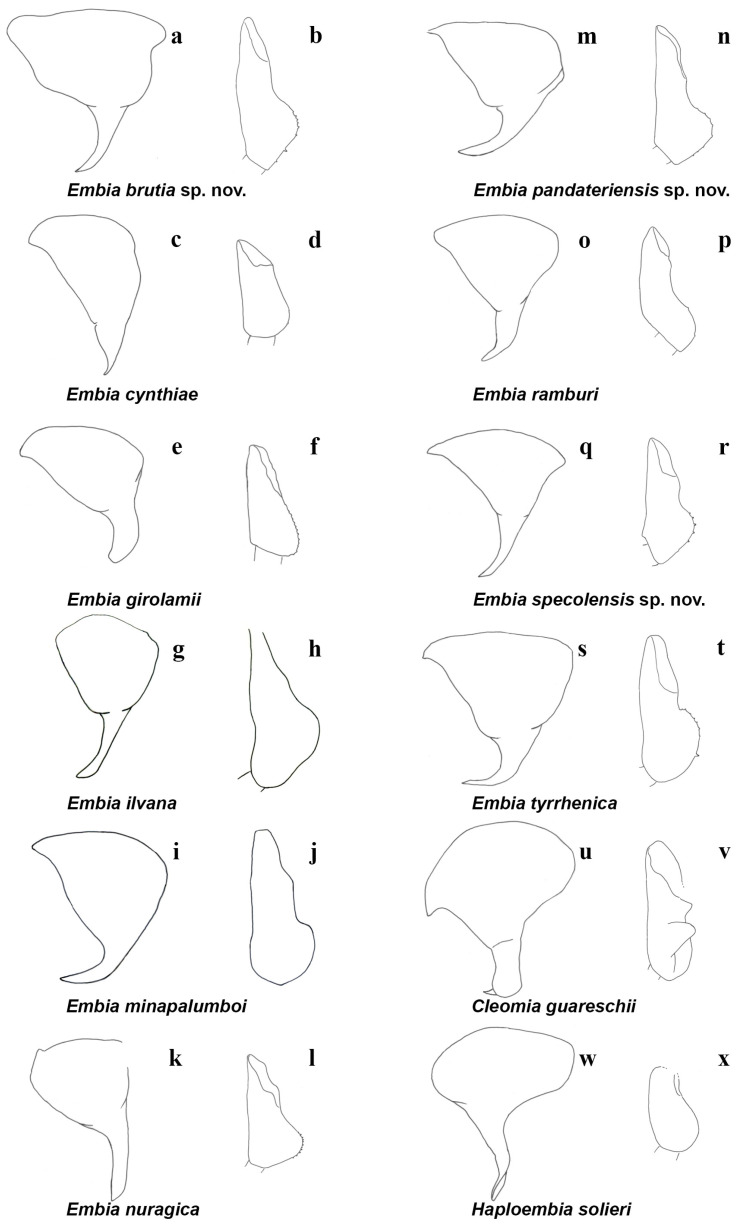
Left hemitergite of the tenth segment (10 L) in the dorsal view (**a**,**c**,**e**,**g**,**i**,**k**,**m**,**o**,**q**,**s**,**u**,**w**) and basal segment of the left cercus (LC_1_) in the dorsal view (**b**,**d**,**f**,**h**,**j**,**l**,**n**,**p**,**r**,**t**,**v**,**x**) of the *Embia* recorded from Italy. Original drawings (**a**,**b**,**g**,**h**,**i**,**j**,**m**,**n**,**o**,**p**,**q**,**r**,**s**,**t**) and redrawn with modifications from Fontana (2002) (**c**,**d**), Fontana (2001) (**e**,**f**), and Ross (1966) (**k**,**l**,**u**,**v**,**w**,**x**) by F. Cianferoni.

## 4. Discussion

The new approach used in this study, i.e., not to clear the specimens in a KOH solution and mount them on slides (see Materials and Methods), was adopted since we realized the importance of the three-dimensionality of pieces of the terminalia (above all, the 10 LP and LPPT P). They need to be observed from different angles. The shape and conformation of the mentioned parts are not noticeable with slide mounting, which allows only dorsal or ventral two-dimensional vision. This is very reductive in understanding the differences among species, which are often totally undetectable, except in cases where obvious differences are understandable without a three-dimensional comparison of the terminalia. In fact, the latest described species are based on evident differences in the terminalia (e.g., *E. girolamii*, *E. cynthiae*), or on different characteristics detectable even bidimensionally (e.g., *E. minapalumboi* showing an unusual shape of hypandrum or *E. ilvana*, which has peculiar mandibles).

However, this new approach makes it possible to compare the various pieces and rotate them to understand and appreciate their three-dimensional conformation and detect differences or similarities among specimens. Furthermore, it is also possible to remove and re-mount the various structures for future comparisons, photos, etc. This is impossible with a definitive preparation and slide mounting, see, e.g., [9], which also prevents the three-dimensional examination of the characteristics of key specimens (such as those of type series). Another advantage of the new method is avoiding (or limiting) possible distortion or alteration of the structures, which could create artifacts and prevent correct comparison (a similar approach has already been used by the authors on other insects with similar challenges, see, e.g., [38]).

However, this should be adopted as an integrative method, which is not always an alternative to examination under a biological microscope. The latter actually allows a higher magnification and optical resolution (albeit with the previously described limitations), often fundamental for interpreting some minute characteristics. However, we recommend not carrying out definitive preparations (especially with type and unique material), which could prevent future comparisons.

In light of this, all the previous data must be re-evaluated. Many of the records in the literature need revision (not only in Italy). Only with a comparative study like this, which includes an additional morphological study on microscopic slides, will it be possible to understand the intraspecific variability of the currently known species correctly and to compare them correctly. An integrated approach, including molecular analyses, will be essential to clarify the relationships.

More generally, the numbers of specimens currently available, especially for some species known based on single specimens, still need to be improved for the correct understanding of the relationships between the various entities. Only with longer series will it be possible to evaluate the validity of the known species correctly. Many of the species described until now (see the Introduction for reference examples) are currently very hard to compare since they are often based on different (sometimes apparently unique) characteristics. At the same time, the terminalia remain difficult to compare due to the limitations mentioned above. It is not excluded that in some cases, in the future, it will be possible to realize that some of the species described so far correspond to extreme forms of a cline or to populations that fall within the variability of the same species.

Even in the case of wing polymorphism, this currently cannot be used as a stable differential characteristic (see, e.g., the key in [31]) because the specimen numbers are too low. More data are needed to understand whether the various species are monomorphic, dimorphic or polymorphic (see, e.g., the case of *E. tyrrhenica* summarized by Fontana et al. [4]).

Currently, the entire taxonomy of these insects is based almost exclusively on the morphology of the terminalia of males. In the case of females and immature specimens, the situation is even more complicated, and a long series and detailed comparative studies on chaetotaxis are essential. However, this remains an open field that needs to be addressed carefully.

It is now evident that the diversity of Embioptera, at least in southern Europe, has been underestimated, and only in recent years are we beginning to understand its richness and complexity [21,22,24,25] and present contribution. In the Mediterranean, until a few years ago, our knowledge was better for North Africa than for Southern Europe, essentially thanks to the key contribution by Ross [18].

A further consideration concerns breeding (*ad libitum*), which could probably affect the size of adults. An indication in this sense could come from the marked difference in size of the paratype females and males of *E. specolensis* sp. nov. (compared with the holotype collected directly in natural conditions) resulting from breeding. To establish this, much more comparative data would be necessary.

## Figures and Tables

**Table 1:** Provisional checklist of the Embioptera in Italy.

Fam. **Embiidae**	
***Cleomia*** Stefani, 1953	
*Cleomia guareschii* Stefani, 1953	Sardinia, Balearic Islands, and Spain
***Embia*** Latreille, 1829	
*Embia brutia* **sp. nov.**	Known only from Calabria (Southern Italy)
*Embia cynthiae* Fontana, 2002	Known only from northern Sardinia
*Embia girolamii* Fontana, 2001	Known only for the northern coast of Tuscany (Central Italy)
*Embia ilvana* Fontana & Forbicioni, 2024	Known only from Elba Island (Central Italy)
*Embia minapalumboi* Fontana, 2024	Known only from the Madonie mountains (Sicily)
*Embia nuragica* Stefani, 1953	Known only from northern Sardinia
*Embia pandateriensis* **sp. nov.**	Known only from Ventotene Island (Central Italy)
*Embia ramburi* Rimsky-Korsakow, 1905	Southern France and Peninsular Italy (confirmed for: Tuscany, Lazio)
*Embia specolensis* **sp. nov.**	Known only from Florence (Central Italy)
*Embia tyrrhenica* Stefani, 1953	Sardinia and Peninsular Italy (confirmed for: Tuscany, Lazio)
Fam. **Oligotomidae**	
***Haploembia*** Verhoeff, 1904	
*Haploembia solieri* Rambur, 1842	Several Mediterranean countries (incl. mainland Italy and islands), Madeira, Canary Islands, Crimea, southern Russia and Caucasus. Introduced in western USA and also recorded from Japan.

## Data Availability

This published work and the nomenclatural acts it contains have been registered in ZooBank—The Official Registry of Zoological Nomenclature. The LSID (Life Science Identifier) for this publication is urn:lsid:zoobank.org:pub:2B6C77F3-F797-40E8-9ED2-CA072C99E4AE. The original contributions presented in this study are included in the article material; further inquiries can be directed to the corresponding author.
